# When Too Much Is Enough: Pediatric Cyproheptadine Overdose with Confirmatory Level

**DOI:** 10.5811/cpcem.2017.2.33313

**Published:** 2017-07-06

**Authors:** Terrance McGovern, Justin McNamee, Steven Marcus, Josh Kashani

**Affiliations:** * St. Joseph’s Regional Medical Center, Department of Emergency Medicine, Paterson, New Jersey; † Emergency Medicine Professionals, Ormond Beach, Florida; ‡ Rutgers University Hospital, Department of Emergency Medicine, Newark, New Jersey

## Abstract

Cyproheptadine is an H-1 antihistamine with anticholinergic and antiserotonergic effects. Cyproheptadine’s most common use has been in the management cold-induced urticaria. It is often used in primary care for its side effect of appetite stimulation. Recently there has been increasing interest in its use in the treatment of drug-induced serotonin syndrome. Cyproheptadine overdose is uncommonly reported in the medical literature. We report the rare case of a pediatric cyproheptadine overdose with a confirmatory cyproheptadine level.

## INTRODUCTION

Cyproheptadine is an antagonist at muscarinic, H-1 histaminic and serotonin receptors.^1^ Previously reported cases of pediatric cyproheptadine toxicity reflect the antimuscarinic nature of the drug with resulting anticholinergic signs and symptoms.^2^,^3^,^4^ Only one prior reported case confirms the exposure with a supporting drug level.^5^ We report a case of symptomatic pediatric cyproheptadine overdose with a confirmatory therapeutic drug level.

## CASE REPORT

A 5-year-old female with no significant past medical history began to experience urinary incontinence, ataxia, confusion, visual and auditory hallucinations while at home with her mother. Shortly thereafter, the patient’s mother found her recently prescribed cyproheptadine bottle empty, which previously contained approximately 15–20 (4 mg) tablets for appetite stimulation. Emergency medical services was contacted and she was transported to the emergency department (ED). Upon arrival her physical examination revealed a blood pressure of 111/89 mmHg, heart rate of 140 beats per minute, respiratory rate of 26 breaths per minute, axillary temperature of 97° F and an oxygen saturation of 100% on room air. On physical exam she was found to be disoriented to person, place, time and situation. While she was confused throughout her ED course, she would follow commands when asked to do so. Additionally, she had ongoing visual and auditory hallucinations during her stay. Her pupils were 3 mm, equal and reactive. The oral mucosa, as well as the axillae, were dry. Bowel sounds were present and her abdomen was soft and non-tender throughout. She had clear lung sounds and aside from the tachycardia a benign cardiovascular exam. No apparent petechiae, purpura or skin abnormalities were noted.

Laboratory evaluation including a complete blood count and basic metabolic profile were within normal limits with the exception of a slightly decreased CO_2_ of 22 mmol/L. Alcohol, acetaminophen and salicylate levels were negative. An electrocardiogram showed sinus tachycardia with a ventricular rate of 151 beats per minute with normal axis and intervals. A urine enzyme-mediated immunotransferase screen for drugs of abuse was negative, and human immunodeficiency screen was also negative. A cyproheptadine level obtained approximately 12 hours after exposure was 0.054 mg/L (therapeutic range 0.02 – 0.1 mg/L). After consultation with the toxicology service the recommendation was for the patient to be admitted overnight for observation and supportive care. She was subsequently admitted to the pediatric stepdown unit and had a progressive improvement in her symptoms. After 24 hours of inpatient observation she was asymptomatic and discharged home.

## DISCUSSION

Cyproheptadine is an antagonist at muscarinic, H-1 histaminic and serotonergic receptors. It is used in the management of serotonin syndrome, migraine prophylaxis, cold-induced urticaria, appetite stimulation and the management of allergic reactions.^6^–^11^ The diverse nature of cyproheptadine allows it to be used for a variety of clinical indications.

Previously reported cases of pediatric cyproheptadine overdose describe patients with various anticholinergic signs and symptoms. Our patient also exhibited signs and symptoms that were consistent with anticholinergic toxicity with the exception of urinary incontinence. Urinary incontinence, although not classically associated with anticholinergic toxicity, may occur as a result of overflow incontinence.^12^

In our patient we obtained a confirmatory level 12 hours after exposure via gas chromatography – mass spectrometry (GC/MS) of 0.054 mg/L (therapeutic range 0.02 – 0.1 mg/L, per hospital’s laboratory). She was not on any additional medications and therefore cross-reactivity was not likely; nor are there any reports of cross-reactivity interfering with quantitative levels in the medical literature. The therapeutic level of cyproheptadine in the setting of obvious toxicity is consistent with the large volume of distribution and largely unknown human pharmacokinetics. The suspected maximum dose consumed by the patient (weight=16.2 kg) was 80mg or 4.94mg/kg. The recommended dose for patients between two- and six-years-old is 0.25 mg/kg/day, which for our patient would have been a total dose of 4.05 mg/day.^13^ Although the ingestion was not witnessed, the history, supporting level and clinical presentation make it the likely etiology for her presentation.

## CONCLUSION

This case highlights the development of cyproheptadine toxicity in the setting of a therapeutic serum level in a pediatric patient. She was managed conservatively and was discharged without further complication. While there has been one previous pediatric case with a confirmatory serum level reported by Yuan et al., the patient’s serum cyproheptadine level was supratherapeutic based on their cited therapeutic range of cyproheptadine concentration.^5^ We believe that this is the first reported case of a pediatric cyproheptadine toxicity with a supporting therapeutic drug level.

CPC-EM Capsule

What do we already know about this clinical entity?

Cyproheptadine is a muscarinic, H-1 histaminic and serotonergic antagonist that can clinically present with an anticholinergic toxicity in the setting of an overdose.

What makes this presentation of disease reportable?

This is the first pediatric ingestion of cyproheptadine that clinically presented with evidence of toxicity despite having a therapeutic serum drug level.

What is the major learning point?

Especially in toxicologic emergencies, clinicians should focus on the clinical signs and symptoms of the patient rather than the quantitative serum levels obtained.

How might this improve emergency medicine practice?

This case highlights the need for clinicians to continue to rely on their physical exam when evaluating patients with suspected toxic overdose.
